# Monte Carlo simulation to optimize polymyxin B dosing regimens for the treatment of Gram-negative bacteremia

**DOI:** 10.3389/fcimb.2025.1533177

**Published:** 2025-02-26

**Authors:** Yingying Yu, Zheng He, Chengcheng Wang

**Affiliations:** ^1^ Department of Pharmacy, Qilu Hospital (Qingdao), Cheeloo College of Medicine, Shandong University, Qingdao, Shandong, China; ^2^ Department of Neurosurgery, Qilu Hospital (Qingdao), Cheeloo College of Medicine, Shandong University, Qingdao, Shandong, China

**Keywords:** polymyxin B, Monte Carlo simulation, Gram-negative bacteria, bacteremia, pharmacokinetics/pharmacodynamics (PK/PD)

## Abstract

**Objective:**

This study aimed to predict and evaluate the efficacy of various polymyxin B dosing regimens for Gram-negative bacteremia using Monte Carlo simulation, with a specific focus on assessing the efficacy in patients receiving continuous renal replacement therapy (CRRT). The goal was to optimize clinical dosing regimens and guide rational polymyxin B use in practice.

**Methods:**

A total of 1,939 Gram-negative bacterial strains were analyzed, collected between April 2019 and December 2021 through the China Bloodstream Gram-negative Pathogens Antimicrobial Resistance and Virulence Surveillance Network (CARVIS-NET). Pharmacokinetic parameters of polymyxin B from existing literature were used to conduct a Monte Carlo simulation based on pharmacokinetic/pharmacodynamic (PK/PD) theory. The probability of target attainment (PTA) and cumulative fraction of response (CFR) were evaluated across various dosing regimens.

**Results:**

The main pathogens of Gram-negative bacteremia were *Escherichia coli*, *Klebsiella pneumoniae, Pseudomonas aeruginosa*, and *Acinetobacter baumannii*, all of which demonstrated high susceptibility to polymyxin B. For pathogens with a minimum inhibitory concentration (MIC) ≤1 mg/L, all regimens achieved PTA >90%. However, when the MIC increased to 2 mg/L, the PTA for the 500,000 IU q12h regimen decreased to 77.53%, and at an MIC of 4 mg/L, none of the dosing regimens achieved a PTA >90%. For *P. aeruginosa* and *K. pneumoniae* with MIC ≤0.5 mg/L, all regimens demonstrated effectiveness. However, at MIC ≥1 mg/L, significant declines in PTA were observed, with the 500,000 IU q12h and 1.25 mg/kg q12h regimens yielding suboptimal outcomes. In CRRT patients, PTA values declined further, particularly against *K. pneumoniae*, raising concerns about potential treatment failure.

**Conclusion:**

Polymyxin B demonstrates high efficacy for Gram-negative bacteremia with MIC ≤1 mg/L. However, efficacy diminishes as MIC increases, particularly for *P. aeruginosa* and *K. pneumoniae*, where 500,000 IU q12h and 1.25 mg/kg q12h regimens may result in suboptimal outcomes. For CRRT patients with *K. pneumoniae* bacteremia, therapeutic drug monitoring and dose adjustments are crucial to mitigate treatment failure risks.

## 1 Introduction

Polymyxins, a class of non-ribosomal cyclic cationic polypeptide antibiotics, were first isolated in 1947 by Japanese researchers from Bacillus polymyxa cultures. The group primarily includes five variants: A, B, C, D, and E, with polymyxin B and colistin (polymyxin E) being the most commonly used agents in clinical practice. Polymyxins exert their antibacterial effect by binding to lipopolysaccharides on the outer membrane of Gram-negative bacteria, leading to leakage of intracellular contents (Zavascki et al., 2007). Although initially approved for clinical use in the 1950s, concerns over nephrotoxicity and neurotoxicity, along with the emergence of more potent antibiotics, led to a decline in their use. However, in recent years, with the global rise in antibiotic resistance, particularly the emergence of carbapenem-resistant Gram-negative bacilli (CRGNB), positioning polymyxins as a last-line treatment for multidrug-resistant Gram-negative infections.

Compared to colistin, polymyxin B has more favorable pharmacokinetic properties and superior renal safety, making it a preferable choice for the treatment of invasive infections (Tsuji et al., 2019). However, significant variability exists in the recommended dosing regimens across countries and clinical guidelines. For instance, the Chinese product label recommends a fixed dose of 500,000 to 1,000,000 IU/day (Shanghai First Biochemical Pharmaceutical Co. L, 2020), while the FDA recommends a weight-based dosing of 15,000–25,000 IU/kg/day in divided doses (U.S. National Library of Medicine, 2020). International guidelines propose a weight-based regimen with a loading dose of 2.0 to 2.5 mg/kg (1mg=10,000 units) followed by a maintenance dose of 1.25 to 1.5 mg/kg every 12 hours (Tsuji et al., 2019).These discrepancies highlight the challenge of establishing an optimal dosing regimens for polymyxin B.

Previous PK/PD studies (Sandri et al., 2013; Nelson et al., 2015) have demonstrated a correlation between increased polymyxin B dosing and improved clinical outcomes. Suboptimal dosing, particularly with polymyxin B <15,000 units/kg/day, has been linked to insufficient serum drug concentrations and inadequate AUC/MIC ratios, often leading to poor outcomes and treatment failure in critically ill patients (Ismail et al., 2018; Xia and Jiang, 2021).Conversely, higher doses (≥200 mg/day) of polymyxin B have been associated with reduced in-hospital mortality, but at the cost of increased nephrotoxicity. Several studies have confirmed that a daily dose of ≥200 mg/day is independently and significantly associated with a high risk of acute kidney injury (AKI) (Elias et al., 2010; Falagas et al., 2021).These findings underscore the need for individualized dosing strategies that account for both efficacy and safety.

Patients undergoing continuous renal replacement therapy (CRRT) represent a particularly complex population. Traditional practices suggest no need for polymyxin B dose adjustments in renal impairment or CRRT. However, recent studies indicate that CRRT increases the clearance of polymyxin B, potentially requiring higher doses to achieve therapeutic targets (Luo et al., 2022).This creates a challenge in selecting the appropriate dosing regimen for these patients.

Monte Carlo simulation analysis is a well-established approach for optimizing antibiotic dosing regimens. Previous studies, such as those by Sandri et al (Sandri et al., 2013), emphasized the importance of initiating therapy with a loading dose to achieve optimal drug exposure. Miglis et al (Miglis et al., 2018)further explored weight-based loading and fixed-dose regimens, demonstrating their potential in balancing efficacy and toxicity. Studies by Yu et al (Yu et al., 2021) and Wu et al. (2021) showed that adjusting polymyxin B doses in patients with renal insufficiency enhances the likelihood of achieving optimal drug exposure. However, studies on optimal dosing regimens for CRRT patients remain sparse, and the existing researches were primarily based on small, localized patient cohorts,often fail to incorporate bacterial resistance data or tailor treatment strategies for different pathogens (Luo et al., 2022; Wang et al., 2022b), which presents certain limitations.

Building on these findings, this study uses Monte Carlo simulation to evaluate different polymyxin B dosing regimens for treating Gram-negative bacteremia, including in CRRT patients, to provide clinical guidance on optimal dosing strategies. By integrating large-scale epidemiological data on bacterial resistance, our approach aims to optimize and personalize dosing strategies for critically ill patients with Gram-negative bacteremia caused by different pathogens, addressing a gap in the current literature and contributing to the development of more precise and effective treatment protocols.

## 2 Materials and methods

### 2.1 Bacterial strains

This study utilized a publicly available dataset from the CARVIS-NET comprising 1,939 Gram-negative bacterial isolates. These isolates were consecutively and non-repetitively collected from individual patients with clinically and laboratory-confirmed bloodstream infections across 21 centers in 20 cities in China between April 2019 and December 2021. All organisms were considered clinically significant based on local hospital criteria and isolated from high-quality specimens of each patient’s first positive blood culture. The dataset includes community-associated and nosocomial bloodstream infections, with community-associated infections accounting for approximately 47.8%. Infection sources were classified based on clinical records into categories such as respiratory tract infections, urinary tract infections, alimentary tract infections, and others. Ethical approval for the initial data collection was obtained from the Human Research Ethics Committee of PUMCH (Ethics Approval Number: HS2755), as reported in the original study (Xi et al., 2022). No additional ethical approval was required for our analysis, as this study solely relies on publicly accessible data.

### 2.2 Antibiotic susceptibility and MIC determination

Antibiotic susceptibility testing for polymyxin B was performed using the broth microdilution method, as recommended by the Clinical and Laboratory Standards Institute (CLSI). The MICs were interpreted according to the CLSI M100-S32 guidelines or the European Committee on Antimicrobial Susceptibility Testing (EUCAST) standards. Specifically, MIC breakpoints for polymyxin B were as follows: For *Enterobacterales* and *Acinetobacter*, MICs ≤2 mg/L were considered susceptible, and MICs >2 mg/L as resistant. For *Pseudomonas* MICs ≤4 mg/L were categorized as susceptible, and MICs >4 mg/L as resistant (EUCAST, 2022). *E. coli* ATCC 25922, *P. aeruginosa* ATCC 27853 and *K. pneumoniae* ATCC 700603 were used as quality controls (Xi et al., 2022).

### 2.3 Polymyxin B PK/PD parameter targets

The PK/PD index for polymyxin B is the ratio of free drug area under the curve to MIC (*f* AUC/MIC) (Sandri et al., 2013; Tsuji et al., 2019).In this formula, AUC is calculated as Dose/CL where Dose represents the administered dose, CL refers to the drug clearance rate, and *f* denotes the fraction of unbound drug. The reference target value for this ratio is ≥10, with a range of 3.5–28.0 (Hanafin et al., 2023). For specific pathogens, the target values are 20.8 for *P. aeruginosa*, 28.0 for *K. pneumoniae*, and 13.9 for *A. baumannii (* Yang et al., 2021).

### 2.4 Polymyxin B dosing regimens

Based on the polymyxin B product insert, relevant guidelines, and common clinical practice, the following intravenous dosing regimens were evaluated. All regimens were administered over at least 1 hour.

1. 500,000 IU q12h;
2. 1,000,000 IU q12h;
3. 1.25 mg/kg q12h;
4. Loading dose: 2 mg/kg over 1 hour, followed by maintenance: 1.25 mg/kg q12h;
5. Loading dose: 2.5 mg/kg over 1 hour, followed by maintenance: 1.5 mg/kg q12h.

### 2.5 Monte Carlo simulation of antimicrobial therapy

#### 2.5.1 Polymyxin B PK parameters

Polymyxin B PK parameters were derived from population pharmacokinetic (PPK) studies in different patient populations. For patients with normal renal function, polymyxin B CL was reported as 0.028 ± 0.007 L/h/kg (Yu et al., 2022), based on adult patients with endogenous creatinine clearance ranging from 60 to 120 mL/min and bloodstream infections caused by carbapenem-resistant *K. pneumoniae*, as detailed in **Table 1**; while in patients undergoing CRRT, polymyxin B CL was slightly higher, measured at 0.033 ± 0.003 L/h/kg (Luo et al., 2022). The PPK analysis for CRRT patients was based on adult intensive care unit (ICU) patients with confirmed or suspected infections caused by carbapenem-resistant organisms. The mean fraction of unbound drug in plasma was 0.42 (range: 0.26–0.64) (Sandri et al., 2013; Zheng et al., 2023).

**Table 1:** Population pharmacokinetic parameters in different patient populations.

Demographic characteristics	Normal Renal Function	CRRT
Age (years)	68 (63-73)	65 (27-93)
Sex (male/female)	7/2	33/16
Weight (kg)	60 (55-65)	60 (41-91)
Creatinine clearance (ml/min)	89 (68–106)	–
CL (L/kg/h)	0.028 ± 0.007	0.033 ± 0.003

CRRT:continuous renal replacement therapy, CL: clearance rate

#### 2.5.2 Monte Carlo simulation method

Monte Carlo simulation were performed using Oracle Crystal Ball (version 11.1.1.4.400) software to evaluate the efficacy of different polymyxin B dosing regimens. A total of 10,000 simulations were conducted, assuming that CL follows a normal distribution and *f* follows a uniform distribution, with a 95% confidence interval. Based on the PK/PD parameter targets, the probability of target attainment (PTA) was calculated for each dosing regimen at various MIC values. Custom MIC values and probability distributions were input into the simulation model, and corresponding PK/PD target values were adjusted. The cumulative fraction of response (CFR) was then calculated to express the expected probability of each dosing regimen achieving the target threshold against a population of pathogens. Generally, dosing regimens with PTA or CFR ≥90% are considered appropriate for empirical antimicrobial therapy.


$$\text{CFR}=\sum_{i=1}^{n}{\text{PTA}}_{\text{i}}\times Fi$$

Among them, PTAi refers to the probability of target attainment for a specific MIC value, and Fi represents the percentage of each MIC distribution within the population of bacterial strains.

## 3 Results

### 3.1 Distribution of MIC for Gram-negative bacteria

Among the 1,939 Gram-negative bacterial strains isolated, 1,724 (88.91%) belonged to the *Enterobacterales* family, while 207 (10.67%) were non-fermentative bacteria. The five most common pathogens identified were *E. coli* (896 strains, 46.21%), *K. pneumoniae* (612 strains, 31.56%), *P. aeruginosa* (95 strains, 4.90%), *A. baumannii* (82 strains, 4.23%), and *E. cloacae* (58 strains, 2.99%) (**Table 2**).

**Table 2:** Distribution of 1,939 Gram-negative bacteremia isolates.

Bacterial Species	Number of Isolates	Proportion (%)
*E. coli*	896	46.21%
*K. pneumoniae*	612	31.56%
*P. aeruginosa*	95	4.90%
*A. baumannii*	82	4.23%
*E. cloacae*	58	2.99%
Other Gram-negative bacteria	196	10.11%
Total	1939	100%

The susceptibility of these Gram-negative bacteria to polymyxin B varied across different infection sources, as shown in **Table 3**. Bloodstream infection isolates derived from catheter-related bloodstream infections exhibited the lowest susceptibility to polymyxin B (90.48%), whereas isolates from alimentary tract and cardiovascular system infections demonstrated 100% susceptibility.

**Table 3:** Susceptibility of Gram-negative bacteria to polymyxin B across different infection sources.

Infection Sources	Number of isolates susceptible to polymyxin B	Total
Respiratory tract infection	313 (91.79%)	341
Urinary tract infection	254 (96.58%)	263
Alimentary tract Infection	51 (100%)	51
Central nervous system infection	15 (93.75%)	16
Liver abscess	80 (97.56%)	82
Biliary tract infection	187 (94.92%)	197
Abdominal infection of other organs (except liver and biliary tract infection)	147 (91.30%)	161
Pelvic infection (including puerperal infection)	16 (94.12%)	17
Skin and soft tissue infection	69 (98.57%)	70
Cardiovascular system infection	5 (100%)	5
Catheter-related bloodstream infections	38 (90.48%)	42
Others	649 (93.52%)	694
Total	1824 (94.07%)	1939

The isolated Gram-negative bacteria demonstrated a high level of susceptibility to polymyxin B, with 94.07% of the isolates exhibiting an MIC ≤2 mg/L. *E. coli* showed the highest susceptibility, with MIC50 and MIC90 values of 0.25 mg/L and 0.5 mg/L, respectively. For *K. pneumoniae*, the MIC50 and MIC90 values were both 0.5 mg/L, while for *P. aeruginosa*, they were both 1 mg/L, and for *A. baumannii*, they were 0.5 mg/L and 1 mg/L, respectively (**Table 4**).

**Table 4:** MIC distribution of polymyxin B against Gram-negative bacteria (%).

Bacteria	MIC (mg/L)										MIC50 (mg/L)	MIC90 (mg/L)
	0.25	0.5	1	2	4	6	16	32	64	128		
*E. coli*	67.41	27.12	3.68	0.33	0.33	0.45	0.22	0	0	0.45	0.25	0.5
*K.pneumoniae*	33.82	57.68	4.25	1.31	0.33	0.82	0.98	0.33	0.16	0.33	0.5	0.5
*P. aeruginosa*	4.21	44.21	48.21	2.11	0	0	1.05	0	0	0	1	1
*A. baumannii*	35.37	52.44	8.54	0	1.22	0	0	1.22	0	1.22	0.5	1
*E. cloacae*	36.21	41.38	1.72	1.72	0	0	0	1.72	1.72	15.52	0.5	128
Total	46.98	39.50	6.24	1.34	0.62	0.62	0.57	0.26	0.31	3.56		

MIC50, Minimum inhibitory concentration required to inhibit the growth of 50% of isolates.

MIC90, Minimum inhibitory concentration required to inhibit the growth of 90% of isolates.

### 3.2 Efficacy of polymyxin B in treating Gram-negative bacteremia in patients with normal renal function

**Figure 1:**
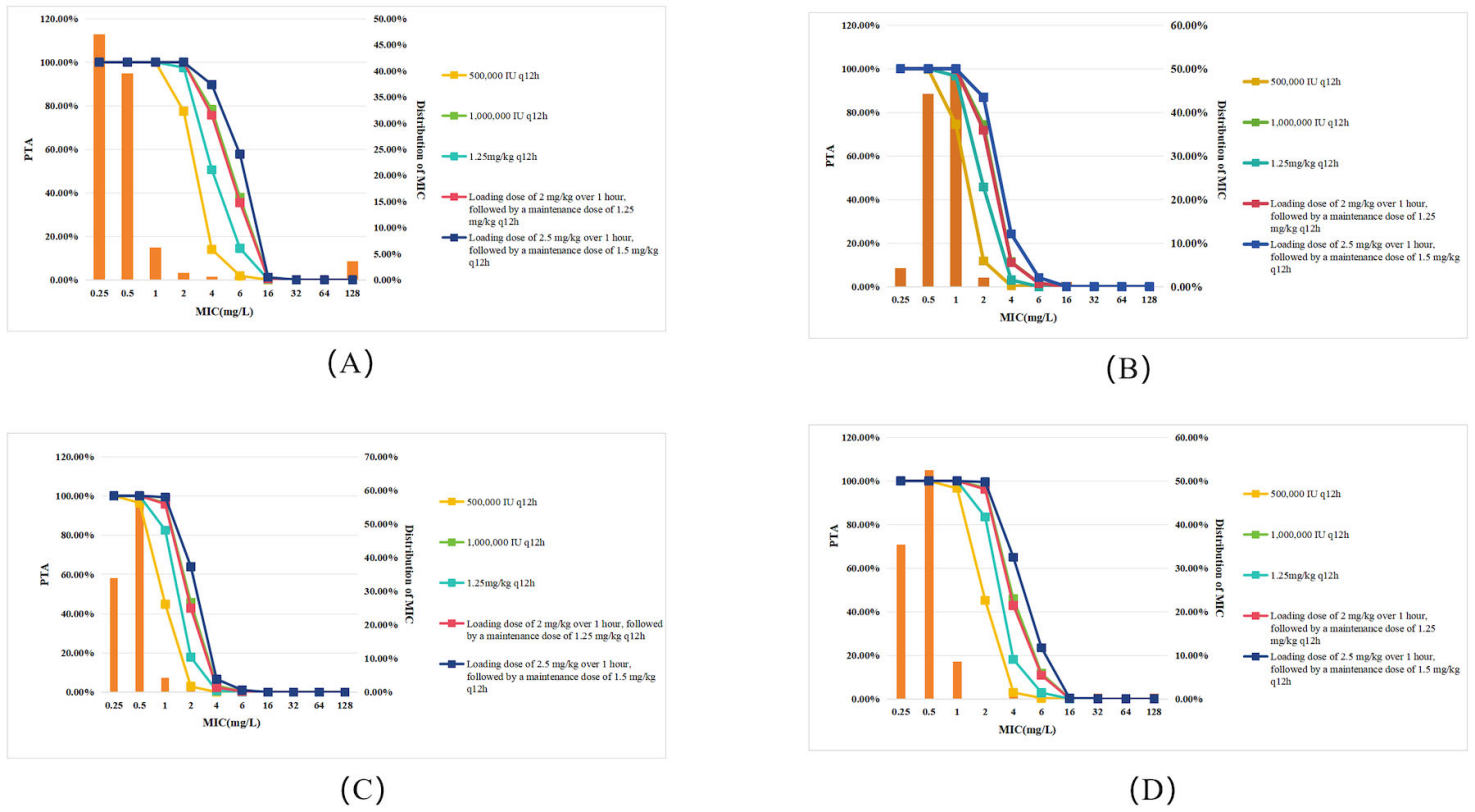
PTA for different polymyxin B dosing regimens against Gram-negative bacteremia in patients with normal renal function. Each panel represents PTA values at varying MICs (mg/L): **(A)** All Gram-negative bacteria; **(B)** *P. aeruginosa;* **(C)** *K. pneumoniae;* **(D)** *A. baumannii.* Legend represent dosing regimens as follows: 500,000 IU q12h (orange); 1,000,000 IU q12h (green); 1.25 mg/kg q12h (deep green); Loading dose 2 mg/kg, maintenance 1.25 mg/kg q12h (red); Loading dose 2.5 mg/kg, maintenance 1.5 mg/kg q12h (deep blue). The bars represent the MIC distribution of polymyxin B against Gram-negative bacteria.

The PTA values of various polymyxin B dosing regimens for treating Gram-negative bacteremia are shown in **Figure 1**. As demonstrated in **Figure 1A**, all dosing regimens achieved PTA ≥90% for Gram-negative bacteremia isolates with MIC ≤1 mg/L. However, when the MIC increased to 2 mg/L, 500,000 IU q12h showed a sharp decline in PTA to 77.53%, falling below the target threshold, and when the MIC increased to 4 mg/L, none of the regimens could achieve a PTA >90%.

For *P. aeruginosa* infections (**Figure 1B**), all dosing regimens achieved PTA ≥90% at MIC ≤0.5 mg/L. However, when the MIC increased to 1 mg/L, 500,000 IU q12h failed to reach the effective target, with PTA dropping to 74.52%. Notably, when MIC increased to 2 mg/L, no regimen achieved PTA >90%.

For *K. pneumoniae* infections (**Figure 1C**), the simulation indicated that when the MIC ≤0.5 mg/L, all regimens reached the target. However, at an MIC of 1 mg/L, the 500,000 IU q12h and 1.25 mg/kg q12h regimens failed to reach the effective target. When the MIC increased to 2 mg/L, none of the regimens achieved a PTA >90%.

For *A. baumannii* infections (**Figure 1D**), the simulation showed that when the MIC ≤1 mg/L, all regimens reached the target. However, at an MIC of 2 mg/L, the 500,000 IU q12h and 1.25 mg/kg q12h regimens failed to reach the effective target. When the MIC increased to 4 mg/L, none of the regimens achieved a PTA >90%.

### 3.3 Efficacy of polymyxin B in treating Gram-negative bacteremia in CRRT patients

**Figure 2:**
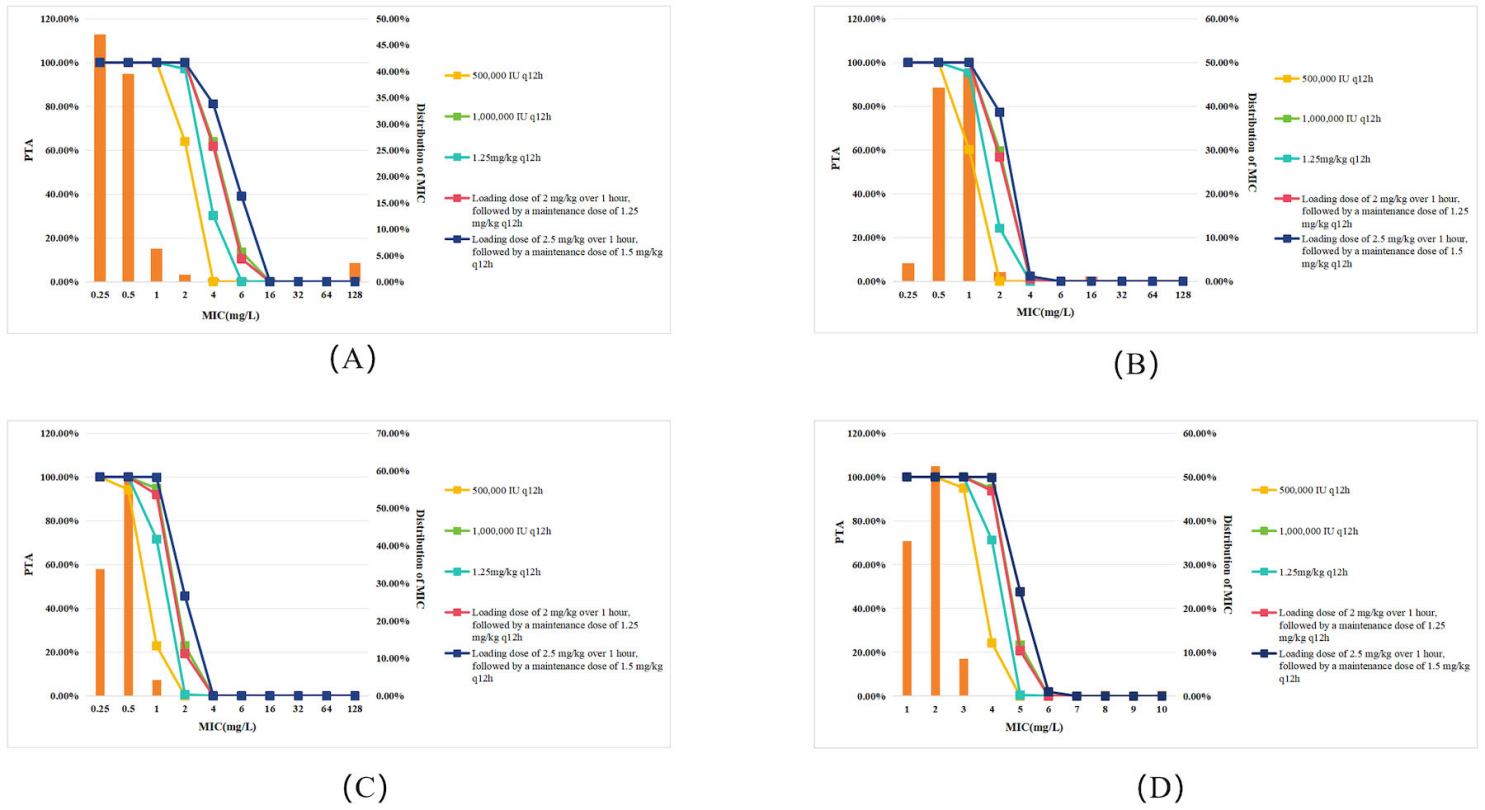
PTA for different polymyxin B dosing regimens against Gram-negative bacteremia in patients undergoing CRRT. Each panel represents PTA values at varying MICs (mg/L): **(A)** All Gram-negative bacteria; **(B)** *P. aeruginosa;* **(C)** *K. pneumoniae;* **(D)** *A. baumannii.* Legend represent dosing regimens as follows: 500,000 IU q12h (orange); 1,000,000 IU q12h (green); 1.25 mg/kg q12h (deep green); Loading dose 2 mg/kg, maintenance 1.25 mg/kg q12h (red); Loading dose 2.5 mg/kg, maintenance 1.5 mg/kg q12h (deep blue). The bars represent the MIC distribution of polymyxin B against Gram-negative bacteria.

Compared to patients with normal renal function, CRRT patients exhibited a decrease in PTA across all dosing regimens and target pathogens (**Figure 2**). As shown in **Figure 2A**, the PTA of 500,000 IU q12h regimen for all Gram-negative bacteria at MIC=2 mg/L declined by approximately 13.6% (from 77.53% to 63.97%) in CRRT patients compared to those with normal renal function (**Figure 1A**). Specifically, for *P. aeruginosa* (**Figure 2B**), the PTA declined from 11.80% to 0.07%; for *K. pneumoniae* (**Figure 2C**), it dropped from 2.88% to 0.01%; and for *A. baumannii* (**Figure 2D**), it declined from 45.26% to 24.21%. However, using a PTA ≥90% as the criterion for regimen selection, CRRT did not significantly impact treatment outcomes.

Considering the higher therapeutic demands for severe infections caused by multidrug-resistant Gram-negative bacteria, PTA was also evaluated with a more stringent≥95% threshold for regimen selection (Paranos et al., 2022). Under this criterion, CRRT patients with *K. pneumoniae* infections (MIC≥0.5mg/L) could not achieve therapeutic efficacy with the 500,000 IU q12h regimen. Furthermore, neither the 1,000,000 IU q12h regimen nor the dosing strategy consisting of a loading dose of 2 mg/kg followed by a maintenance dose of 1.25 mg/kg q12h was effective when MIC ≥1mg/L. Similarly, for *A. baumannii* infections, both the 1,000,000 IU q12h regimen and the aforementioned loading and maintenance dose strategy failed to achieve the effective target when MIC≥2mg/L.

### 3.4 CFR values of polymyxin B dosing regimens for Gram-negative bacteremia

The CFR values for different polymyxin B dosing regimens in treating Gram-negative bacteremia in patients with normal renal function are shown in **Table 5**. The Monte Carlo simulation indicated that all simulated regimens achieved a CFR >90% for Gram-negative bacteremia, suggesting good clinical efficacy. However, when the pathogen was *P. aeruginosa*, the 500,000 IU q12h regimen had a CFR value of 85.12%, indicating a potential for treatment failure.

**Table 5:** CFR of polymyxin B dosing regimens for treating bacteremia in patients with normal renal function.

Pathogen Category	CFR(%)				
	500,000 IU q12h	1,000,000 IU q12h	1.25 mg/kg q12h	Loading dose 2 mg/kg, maintenance dose 1.25 mg/kg q12h	Loading dose 2.5 mg/kg, maintenance dose 1.5 mg/kg q12h
All Gram-negative bacteria	**93.82**	**94.83**	**94.24**	**94.42**	**94.77**
*P. aeruginosa*	85.12	**98.28**	**96.36**	**98.37**	**98.78**
*K. pneumoniae*	**91.63**	**96.29**	**95.22**	**96.17**	**96.52**
*A.baumannii*	**96.27**	**96.84**	**96.46**	**97.13**	**97.16**

CFR values ≥90% (bold) are considered optimal for empirical therapy.

In CRRT patients, all regimens showed a decrease in CFR compared to those with normal renal function (**Table 6**). However, using CFR ≥90% as the criterion for regimen selection, CRRT only affected the treatment of *K. pneumoniae*. For CRRT patients with *K. pneumoniae* infections, the CFR for the 500,000 IU q12h regimen dropped to 89.59%, indicating a potential risk of suboptimal treatment.

**Table 6:** CFR values of polymyxin B dosing regimens for treating bacteremia in CRRT patients.

Pathogen Category	CFR(%)				
	500,000 IU q12h	1,000,000 IU q12h	1.25 mg/kg q12h	Loading dose 2 mg/kg, maintenance dose 1.25 mg/kg q12h	Loading dose 2.5 mg/kg, maintenance dose 1.5 mg/kg q12h
All Gram-negative bacteria	**93.58**	**94.52**	**94.04**	**94.40**	**94.59**
*P. aeruginosa*	77.31	**98.15**	**94.78**	**97.95**	**98.52**
*K. pneumoniae*	89.59	**95.69**	**94.43**	**95.55**	**96.48**
*A. baumannii*	**96.06**	**96.78**	**96.41**	**96.85**	**96.92**

CFR values ≥90% (bold) are considered optimal for empirical therapy.

## 4 Discussion

With the widespread use of carbapenem antibiotics, CRGNB, which have emerged under the selective pressure of powerful antibiotics, have become a major global public health threat. Compared to other infections, bloodstream infections caused by CRGNB have a significantly higher mortality rate. A study by Falcone et al. (2023) showed that the 30-day mortality rate for bloodstream infections caused by carbapenem-resistant bacteria (26.6%~43.2%) was significantly higher than that for infections caused by carbapenem-sensitive bacteria (13.7%).

Treatment options for CRGNB are extremely limited, with sensitive drugs including tigecycline, polymyxins, and ceftazidime-avibactam. Polymyxin B has high sensitivity against common Gram-negative bacteria. In this study, the sensitivity rate of polymyxin B against common Gram-negative bacteria ranged from 96.3% to 99.0%, consistent with another surveillance report showing sensitivity rates between 95.1% and 99.3% (Liu and Ji, 2024). Even for carbapenem-resistant strains, polymyxin B exhibited high efficacy, with sensitivity rates of 89.7%, 88.3%, 95.0%, and 99.0% for carbapenem-resistant *E. coli*, *K. pneumoniae*, *A. baumannii*, and *P. aeruginosa* respectively. Despite its high sensitivity, several factors, including the expression of antimicrobial activity, outdated drug labels, and uncertainties in susceptibility testing, have contributed to inconsistent clinical application of polymyxin B (Tsuji et al., 2019). Therefore, determining the appropriate dosing strategy is a key aspect of optimizing polymyxin B treatment regimens.

In our study, polymyxin B effectively treated Gram-negative bacteremia when the MIC was ≤1 mg/L. However, the effectiveness of the treatment regimen decreased when the infecting pathogens were *P. aeruginosa* or *K. pneumoniae*. At an MIC of 1 mg/L, the 500,000 IU q12h may result in suboptimal clinical outcomes. The CFR values further indicate that the 500,000 IU q12h carries a significant risk of treatment failure, especially in CRRT patients. This may be attributed to the lower sensitivity of *P. aeruginosa* to polymyxin B compared to other bacteria and the higher target values for *K. pneumoniae*. To optimize targeted therapy for these pathogens, higher off-label doses should be considered to ensure effective treatment. However, when the MIC increases to 2 mg/L, none of the simulated dosing regimens can achieve the PTA target (≥90%) for bloodstream infections caused by *P. aeruginosa* or *K. pneumoniae*. According to EUCAST (2022) guidelines, the susceptibility breakpoint for polymyxins is 2 mg/L for *K. pneumoniae*, indicating that, in clinical practice, susceptibility testing may indicate sensitivity, but the clinical treatment outcomes might still be poor.It is important to note that the results of the Monte Carlo simulation are highly dependent on the input parameters and bacterial susceptibility. *f* AUC/MIC is considered the best PK/PD index for predicting the efficacy of polymyxin B, but there remains no consensus on the recommended target values, and the target values vary significantly across studies, leading to differences in study outcomes. For instance, Wang et al (Wang et al., 2022a)proposed a target of 50 and concluded that in elderly patients with multidrug-resistant Gram-negative bacterial infections, doses of 50 mg and 75 mg q12h achieve an optimal balance between nephrotoxicity and efficacy. Yu et al. (2022) adopted bacteria-specific target values and found that for isolates with MIC ≤1 mg/L, a maintenance dose of 1 mg/kg q12h could achieve a PTA >90%. In our study, we selected an *f*AUC/MIC target of 10, which is the average value required to reduce bacterial counts by 1 log10 in a mouse thigh infection model for nine Gram-negative bacteria. For *K. pneumoniae*, *P. aeruginosa*, and *A. baumannii*, the selected targets were the doses required to reduce bacterial counts by 1 log10, which were 28.0, 20.8, and 13.9, respectively (Landersdorfer et al., 2018; Society of Clinical Microbiology and Infection of China International Exchange and Promotion Association for Medical and Healthcare CMGotLMSotCMA and Clinical Microbiology Group of the Microbiology and Immunology Society of the Chinese Medical Association, 2020).

Critically ill patients often exhibit substantial pathophysiological changes, such as hepatic and renal dysfunction, hypoalbuminemia, extensive fluid resuscitation, and alterations in drug distribution volume, all of which can significantly impact the PK of antibiotics. In this study, the mean unbound fraction of polymyxin B was assumed to be 0.42, based on data derived from ICU patients. However, actual unbound fractions can vary considerably across individual patients, particularly in critically ill populations, potentially influencing the interpretation of therapeutic outcomes.

Polymyxin B is predominantly cleared through non-renal pathways, with less than 1% of the drug excreted unchanged in the urine (Zavascki et al., 2007). Consequently, dose adjustments are generally unnecessary in patients with renal insufficiency or those undergoing renal replacement therapy (RRT). However, CRRT, a vital intervention in critically ill patients, can significantly alter drug clearance, subsequently affecting PK/PD parameters and therapeutic outcomes. Evidence suggests that in CRRT patients, especially those receiving continuous venovenous hemodiafiltration (CVVHDF), polymyxin B clearance may increase (Luo et al., 2022; Hanafin et al., 2023; Pi et al., 2023). A study by Luo et al. (2022) showed that the clearance of polymyxin B in CRRT patients (1.95 L/h) was higher than in non-CRRT patients (1.5 L/h). Hanafin et al. (2023) reported that in CVVHDF patients, the steady-state AUC_0-24h_ was 50% lower than in patients not receiving CRRT, underscoring the need to consider the impact of CRRT on therapeutic outcomes. Consistent with these observations, our study demonstrated that CRRT patients exhibited a reduced PTA for polymyxin B across multiple dosing regimens compared to non-CRRT patients. When using PTA or CFR ≥90% as the threshold for therapeutic effectiveness, CRRT had no significant impact on treatment outcomes except for *K.pneumoniae* infections. However, when applying the stricter criterion of PTA ≥95%, a notable shift in efficacy was observed. In CRRT patients, both the 1,000,000 IU q12h regimen and the dosing strategy of 2 mg/kg loading dose followed by a maintenance dose of 1.25 mg/kg q12h demonstrated reduced efficacy against *K.pneumoniae* and *A.baumannii*, particularly when the MIC values ranged from 0.5 to 2 mg/L. Therefore, for these patients, it is recommended to enhance TDM and adjust drug dosing based on the monitoring results to ensure treatment efficacy and minimize the risk of resistance.

Due to the limited sample size of bloodstream infections caused by carbapenem-resistant bacteria, the bacterial susceptibility data used in this study are based on bloodstream infections caused by all Gram-negative bacteria. Since polymyxin B is primarily used for infections caused by carbapenem-resistant bacteria, differences in sensitivity to polymyxin B may lead to varying clinical outcomes. Data from the China Bacterial Resistant Investigation Collaborative System(BRICS) showed (Liu and Ji, 2024) that the resistance rate of *E. coli* to polymyxin B is 1.6%, while the rate increases to 10.3% in carbapenem-resistant *E. coli*. For *K. pneumoniae*, the resistance rate of to polymyxin B is 4.5%, rising to 11.7% in carbapenem-resistant strains. The resistance rate of *P. aeruginosa* is 0.7%, while the carbapenem-resistant strain has a slightly higher rate of 1%. Similarly, for *A. baumannii*, the resistance rate of to polymyxin B is 4.9%, increasing slightly to 5.0% in carbapenem-resistant strains. Susceptibility testing results showed that carbapenem resistance significantly affected the sensitivity of *E. coli* and *K. pneumoniae* to polymyxin B, but had a smaller impact on *P. aeruginosa* and *A. baumannii*. Therefore, a more conservative approach should be taken when interpreting CFR values for *E. coli* and *K. pneumoniae.*

The safety of polymyxin B is also an important consideration when optimizing dosing regimens. Nephrotoxicity is the main dose-limiting toxicity of polymyxin B, affecting up to 30% of patients (Phe et al., 2014; Rigatto et al., 2015; Oliota et al., 2019). A study by Elias et al., 2010 showed that a polymyxin B dose ≥200 mg/day was associated with a higher incidence of severe renal injury. Our study showed that as the drug dose increased, the probability of achieving the target concentration also increased, but higher doses were associated with an increased risk of severe renal injury. Weight-adjusted dosing regimens may result in suboptimal drug concentrations in underweight patients (Miglis et al., 2018; Hanafin et al., 2023), or significant adverse reactions in overweight patients (Wang et al., 2022a). Our study showed that when the MIC of the pathogen was ≤0.5 mg/L, a fixed-dose regimen of 500,000 IU q12h had similar efficacy to weight-adjusted dosing regimens, with potentially better safety. When the MIC increased to 1 mg/L, the 1,000,000 IU q12h regimen had similar efficacy to the loading-dose plus maintenance-dose regimen, but potentially better safety. A fixed-dose regimen may be the better option after balancing efficacy and adverse reactions, but further studies are needed to determine the optimal regimen.

Although previous studies have explored the optimization of polymyxin B dosing regimens for the treatment of Gram-negative infections, these studies often focus on single-pathogen infections or lack considerations for the dynamic changes in critically ill patients, particularly those receiving CRRT. Furthermore, previous studies typically did not incorporate comprehensive bacterial resistance data or explore individualized treatment strategies for different pathogens. Our study addresses these gaps by combining large-scale epidemiological resistance data with Monte Carlo simulations to optimize polymyxin B dosing regimens for various Gram-negative pathogens, including those in CRRT patients. However, this study has several limitations that should be acknowledged. First, all polymyxin B dosing regimens in the simulation were based on monotherapy, without accounting for the potential synergistic effects or improved clinical outcomes associated with combination therapy. The rapid spread of plasmid-mediated MCR-1 resistance genes has significantly impacted the effectiveness of polymyxin antibiotics (Liu et al., 2016). Combination antibiotic therapy presents an attractive option for treatment. Additionally, factors such as the safety of high-dose polymyxin B administration, the presence of heteroresistance, and the link between colistin resistance and increased in-hospital mortality further highlight the advantages of combination therapy (Tsuji et al., 2019). However, our study did not explore the role or impact of combination regimens, which may limit the applicability of the findings in clinical practice. Additionally, the population pharmacokinetic data used in this study were derived from a relatively small cohort, necessitating further data collection and validation to improve model accuracy. Finally, it is important to note that critically ill patients often exhibit significant variability in PK/PD parameters due to dynamic physiological changes, which should be carefully considered when applying these results to clinical practice.

## 5 Conclusion

Our study showed that polymyxin B demonstrated good clinical efficacy for Gram-negative bacteremia, especially in pathogens with lower MICs. However, for infections caused by *P. aeruginosa* and *K. pneumoniae* with higher MIC values, lower-dose regimens, such as 500,000 IU q12h, may result in suboptimal treatment outcomes. In patients undergoing CRRT, both PTA and CFR values decrease across all dosing regimens compared to those with normal renal function, with a notable reduction in efficacy against *K. pneumoniae*. These findings highlight the importance of adjusting dosing regimens based on renal function and MIC values to optimize clinical outcomes. We recommend the routine measurement of MICs and individualized therapy to ensure effective treatment. Furthermore, integrating PK/PD modeling with local resistance patterns and TDM can assist clinicians in selecting the most appropriate antibiotic regimens for Gram-negative bacteremia, especially in complex patient populations such as those receiving CRRT.

## Data Availability

The original contributions presented in the study are included in the article/supplementary material. Further inquiries can be directed to the corresponding author.
